# Species coexistence in a changing world

**DOI:** 10.3389/fpls.2015.00866

**Published:** 2015-10-14

**Authors:** Fernando Valladares, Cristina C. Bastias, Oscar Godoy, Elena Granda, Adrián Escudero

**Affiliations:** ^1^Departamento de Biogeografía y Cambio Global, Museo Nacional de Ciencias Naturales, Consejo Superior de Investigaciones Científicas, Madrid, Spain; ^2^Departamento de Ciencias, Universidad Rey Juan Carlos, Móstoles, Madrid, Spain; ^3^Instituto de Recursos Naturales y Agrobiología de Sevilla, Consejo Superior de Investigaciones Científicas, Seville, Spain; ^4^Laboratoire Ecologie Systématique et Evolution, Université Paris Sud/Centre National de la Recherche Scientifique/AgroParisTech, Université Paris-Saclay, Orsay, France

**Keywords:** competition, facilitation, global change, functional traits, heterogeneity, intraspecific variability, climate change

## Abstract

The consequences of global change for the maintenance of species diversity will depend on the sum of each species responses to the environment and on the interactions among them. A wide ecological literature supports that these species-specific responses can arise from factors related to life strategies, evolutionary history and intraspecific variation, and also from environmental variation in space and time. In the light of recent advances from coexistence theory combined with mechanistic explanations of diversity maintenance, we discuss how global change drivers can influence species coexistence. We revise the importance of both competition and facilitation for understanding coexistence in different ecosystems, address the influence of phylogenetic relatedness, functional traits, phenotypic plasticity and intraspecific variability, and discuss lessons learnt from invasion ecology. While most previous studies have focused their efforts on disentangling the mechanisms that maintain the biological diversity in species-rich ecosystems such as tropical forests, grasslands and coral reefs, we argue that much can be learnt from pauci-specific communities where functional variability within each species, together with demographic and stochastic processes becomes key to understand species interactions and eventually community responses to global change.

## Introduction

**FIGURE 1:**
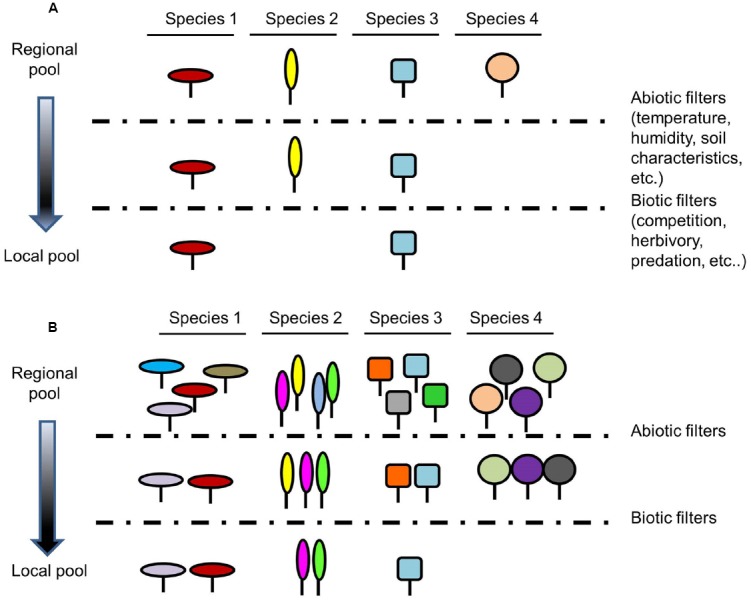
**Influence of intraspecific variability in the filtering of potential species integrating a community. (A)** classical community assembly theory without taking into account intraspecific variability and **(B)** community assembly theory incorporating intraspecific variability. Species with mean trait values matching the abiotic requirements and being either ecologically different or capable of tolerating competition will contribute to the eventual community. By incorporating intraspecific variability, more species will pass biotic and abiotic filters because they are able to adjust by phenotypic plasticity or simply because they are genetically variable so more species could join the community in **(B)** than in **(A).** Each shape represents a species and each color represents a given trait value within a species. Dashed lines represent abiotic and biotic filters.

Species composition of a local community is the result of several processes and factors that act at different scales, none of them being mutually exclusive. This encompasses from features and processes that act at global and regional scales, such as randomness, historical patterns of speciation, extinction, migration as well as dispersal processes, to abiotic factors (physical constraints of the environment) and biotic interactions (both positive and negative) that act at local scale. These factors, known as hierarchical filters, act from broad to fine spatial scales to impose rules on community assembly (Götzenberger et al., 2012). There are numerous theories about these filters and the coexistence mechanisms involved in the composition of species in a community. In this article we focus on those acting at local scales (Figure 1), but we also refer to broader scales and the corresponding interactions since they are key to understand regional and global species diversity.

Biological diversity is about species interactions inter alia, and it is commonly limited by competitive exclusion and sometimes fueled by positive relationships. Competitive exclusion has a crucial role in structuring communities and has therefore prompted intensive ecological research over decades (Pianka and Horn, 2005). Competition has both an evolutionary and an ecological role since it increases diversity through speciation (Brännström et al., 2012) and regulates species diversity through species interactions (Chesson, 2000). Classical coexistence theories establish that each species inhabits a particular niche, involving a given combination of abiotic and biotic factors, where it outcompetes the rest of the species in the local pool (i.e., niche theory; Grinnell, 1917; Gause, 1934). Under this premise, niche overlap penalizes worse competitors, which results in their exclusion from a community, and supports that species coexist by being functionally different and by exploiting different niches (Hutchinson, 1959). If true, the total number of species in an ecosystem is thought to be proportional to the total range of the environment divided by the niche breadth of the species (MacArthur and Levins, 1967). In contrast, neutral theory (Hubbell, 2001) assumes that individuals and species are ecologically interchangeable and therefore equivalent in their competitive ability, i.e., none of the species shows an advantage or disadvantage over the others. According to the neutral theory, random processes, stochastic events, and equivalence between opposite forces are the drivers of population dynamics and species coexistence (Bell, 2000; Hubbell, 2001, 2005; Götzenberger et al., 2012). However, these theoretical frameworks seem insufficient to explain species coexistence in many natural ecosystems and numerous discrepancies have been found between theoretical predictions from classic niche theory and empirical studies (Nathan et al., 2013).

Here we review the theory about the mechanisms underlying the maintenance of species coexistence. Although conclusions and main concepts apply to all sort of living organisms, we have placed special focus on plant communities and, hence, on plant species coexistence and diversity. We give special attention to concepts like competition, facilitation, ecological differences among species, intraspecific variability and environmental heterogeneity. In each section, we discuss how global change may affect species coexistence through modifications in important biotic and abiotic factors. The consideration of all global change factors potentially affecting coexistence would largely exceed the limits of this article so we have focused on the best studied ones and on those illustrating different responses and cascade effects on community dynamics and species interactions. We include an analysis of biological invasions, as a large and unique ecological and evolutionary experiment of coexistence. Also, we encompass the particular case of species coexistence in pauci-specific systems, which complement the better studied cases of tropical, hyperdiverse systems.

## Competition and Related Mechanisms to Explain Species Coexistence

A number of alternatives have been proposed to explain coexistence and diversity when classic niche theory fails (Barot, 2004; Wildová et al., 2012). Under this emergent scenario, classic ideas on competition are being reshaped in a more mechanistic framework giving new perspectives that reconcile neutral and niche theories (Adler et al., 2007), often treated as mutually exclusive explanations.

**FIGURE 2:**
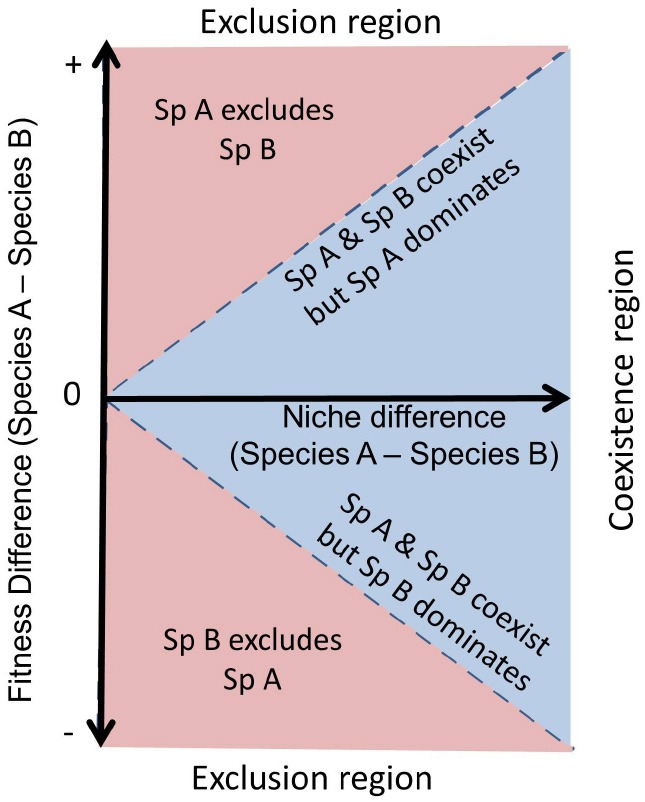
**A theoretical scheme of coexistence and competitive exclusion between two species.** If niche differences between competitors are greater than their fitness asymmetries then both species will show stable coexistence (blue region). In contrast, if fitness differences are greater than niche differences, then the species with higher fitness will exclude the other (red region). Fitness differences also determine which species dominates under stable coexistence. Figure adapted from MacDougall et al. (2009).

This new mechanistic framework is explicitly addressed by combining the two concepts of Chesson’s (2000) framework: the so-called “niche differences” and “fitness differences.” Note that fitness is used as an ecological term, referring to the average competitive ability of a species, and not in an evolutionary context. Although complementing niche theory, niche differences do not determine the outcome of interactions alone. They are only a stabilizing mechanism favoring coexistence by limiting species abundance when they rise to dominance and buffering them against exclusion when they become rare (Adler et al., 2007). Differences in fitness favor dominance, and, in the absence of niche differences, they determine the species that exclude the rest. The key message of Chesson’s (2000) framework is that the outcome of species interaction is jointly determined by the relative strength of niche differences versus fitness differences between species. In this context, coexistence will be fostered when niche differences overcome fitness differences (Figure 2).

This conceptual framework is now raising new questions among ecologists. For instance, since plants have a finite number of potentially limiting resources, the chances to overlap in their niches are in principle rather high (Wildová et al., 2012), particularly when compared to other biological groups such as animals. Is species coexistence therefore maintained in plants because small niche differences overcome small fitness differences or are high levels of niche differentiation still needed? Moreover, many species are dominant or even exclude the rest of the species at a given location, whereas they are inferior competitors at other locations. This opens the question as to what extent are spatially and temporally heterogeneous landscapes together with a large intraspecific variation in functional traits more important for the maintenance of species diversity than average species features and interactions. Giving responses to this kind of questions can undoubtedly advance our basic understanding of species coexistence. But equally important, they can also serve to predict how biological diversity will face a globally changing world.

Whether or not global change drivers are promoting differences among species in niche availability, in competitive ability or in a combination of both is crucial for understanding the evolution of plant communities in terms of diversity and coexistence as well as in terms of ecosystem functioning (Table 1). As a straightforward rule and because niche differentiation tends to stabilize coexistence, species diversity and niche diversity would tend to be correlated as classic niche theory proposes. However, when considering fitness differences possible complex changes may occur. For instance, the reduced competitive ability of the dominant plant species due to lower precipitations during spring (Clark et al., 2011), or due to the interactive effect of rainfall variability with soil pathogens (Gómez-Aparicio et al., 2012) can be dramatic for the affected species up to the point of their extinction at local scales. However, by eliminating the dominant species, rare species could persist, resulting in a community with increased diversity as Mariotte et al. (2013) showed in a drought experiment in grasslands of central Europe. In the same way, increases in fitness instead of reductions would also produce dominance of a single group of species, thereby reducing diversity, as it is the case for the interactive effect of climate change and biological invasions (Vitousek et al., 1997). However, diversity can be increased by equalizing fitness differences if the increase in fitness is for the inferior competitors (Gilman et al., 2010). Extinction of dominant species under extreme events or under intense pressure of global change drivers is very unusual since there are many mechanisms by which dominant species can persist with minimal community changes (Lloret et al., 2012). Although this has been less often reported, changes in species fitness can also reduce fitness differences among competitors reducing the likelihood of competitive exclusion. For example, at the edge between alpine and subalpine vegetation, climate warming is decreasing species fitness of the alpine species but increasing the fitness of the subalpine ones, resulting in an increased diversity at the ecotone (Parolo and Rossi, 2008). Atmospheric CO_2_ enrichment can directly affect species interactions by increasing the fitness of species able to accelerate their growth rates in such enriched atmospheres, but there are still many knowledge gaps on such effects (Busch, 2015).

**TABLE 1:** **Effects of global changes drivers on the outcome of species interaction through their effect on niche and fitness differences**.

**Global change driver**	**Effect on niche differences**	**Effect on fitness differences**	**Examples**
Climate change	Increased climate variability can increase niche differentiation by promoting species with contrasted phenotypes.	New climate regimes possibly change the species hierarchy according their competitive ability. Dominant species become less competitive and subordinate species increase their dominance.	Sherry et al. (2007), Willis et al. (2008), Angert et al. (2009), Mariotte et al. (2013)
Nutrient pollution	Increase in nutrients (N, P) is reducing environmental heterogeneity and thus the chances of species to exploit resources from different niches.	A few species are benefiting from these more homogeneous environments leading to a few species outcompeting the rest. Other species are excluded because they cannot tolerate the new environmental conditions.	Reich et al. (2001), Stevens et al. (2004), Wookey et al. (2009)
Land use change	Novel ecosystems and intense landscape transformations is homogenizing the environment and reducing niche differences within communities. Among communities, land conversion is producing contrasting novel habitats increasing niche differentiation among species at large geographical scales.	Similar effects to nutrient application to agricultural systems. The competitive ability of a few species is dramatically increased, while other species are not able to survive. This reduces the diversity among and within communities.	Hobbs et al. (2006)
Biological invasions	Exotic species with contrasted phenotypes are able to exploit different resources increasing niche differentiation with respect to the resident community. Exotic species with similar phenotypes would reduce niche differentiation and increase niche overlap.	Although most of the introduced species fail to survive and invade because they cannot tolerate the new environmental conditions where they are introduced, successful invaders tend to possess traits that maximize competitive ability for a given quantity of resources.	Strauss et al. (2006), Funk et al. (2008), MacDougall et al. (2009), van Kleunen et al. (2010), Fridley (2012), Godoy and Levine (2014)

The discussion of the impact of global change on species persistence can also be extended to species abundances. Even minor changes in the mechanisms and processes determining coexistence can result in a great impact on species abundances as revealed by simulations based on microorganism traits and demography (Fox, 2012). Dominant or abundant species may exhibit large changes in their abundances despite small niche differences as a consequence of many stabilizing processes operating at different time and spatial scales (Lloret et al., 2012; Yenni et al., 2012). Thus, high competitive ability does not necessarily confer high abundance, particularly under changing or patchy environmental conditions, and even very small niche differences can dwindle the theoretical correlation between adaptive traits and abundance (Fox, 2012).

## Facilitation

Ecological research has mainly focused on competition when referring to species interactions and coexistence. Fitness differences, commonly related not only to the ability to produce offspring but also to the response to competition, reflect the net effect of competition and interspecific facilitation, with coexistence being prompted by an increase in fitness of rare, benefited species. Indeed, facilitation has been widely recognized in recent decades to be an important mechanism for maintaining community diversity and structure, particularly in plant communities (Callaway, 2007). Bruno et al. (2003) integrated facilitation into the niche theory highlighting its potential to increase the realized niche of the species. More recently, McIntire and Fajardo (2014) detailed in an extensive review the mechanisms by which facilitation may increase diversity and coexistence, including (1) stress amelioration, (2) novel habitat creation, (3) increased habitat complexity (i.e., heterogeneity) for a given area, (4) increased access to resources, and (5) service sharing such as pollination or dispersal efficiency.

**FIGURE 3:**
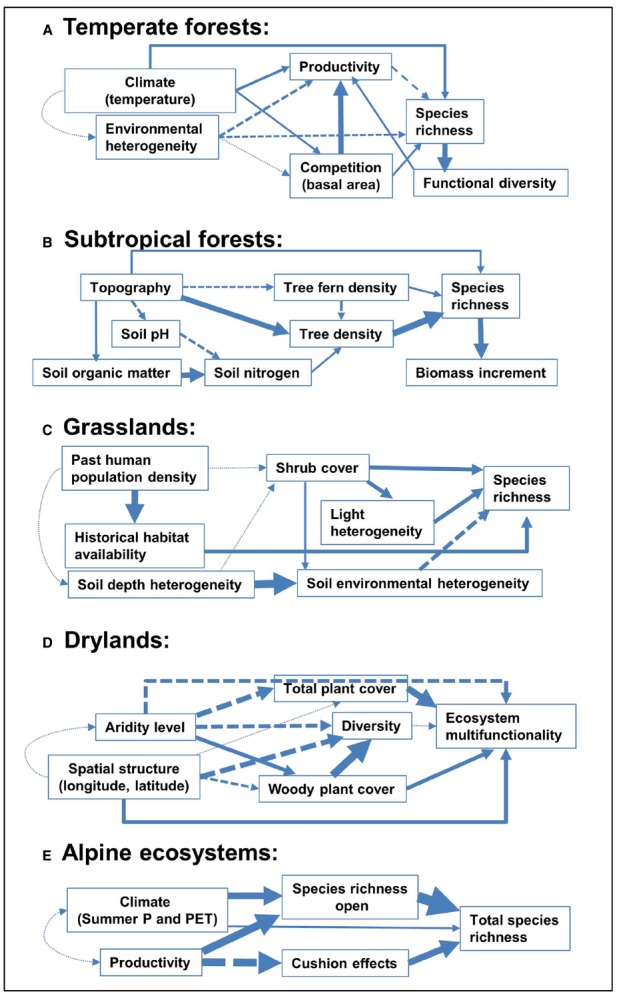
**Representation of direct and indirect pathways relating abiotic and biotic factors with diversity.** We show examples of five study systems, corresponding to **(A)** temperate forests, modified from Paquette and Messier (2011), **(B)** tropical forests, modified from Yasuhiro et al. (2004), **(C)** grasslands, modified from Gazol et al. (2012), **(D)** drylands, modified from Soliveres et al. (2014), and **(E)** alpine ecosystems, modified from Cavieres et al. (2014). Single arrows represent causal paths, where thickness is proportional to the path coefficient (solid: positive, broken: negative, dotted: non-significant). Interlinked influences of landscape conditions and local environmental factors are explaining species richness in contrasted biomes such as subtropical forests and temperate grasslands. However, diversity and coexistence are usually dependent on distinct factors in each biome (i.e., competitive exclusion is more relevant in temperate forests, whereas facilitation mediated by woody cover or cushion effects are more important in drylands and alpine ecosystems, respectively).

Indeed, failure to incorporate these positive interactions likely limits our understanding of ecosystem functioning and responses to climate change (see Brooker et al., 2008, for a review). Positive interactions are thought to increase in importance when environmental conditions are harsher (see examples in Figure 3) becoming, thus, potentially more intense under current and future global changes (Michalet et al., 2006). This increase has been found in alpine and arctic habitats, where plant performance is limited by cold temperatures (Cavieres et al., 2014); in Mediterranean ecosystems subjected to intense and frequent drought events, and in other systems where survival or growth are limited by pervasive strong winds or excessive irradiance (Gómez-Aparicio et al., 2005; Cavieres and Badano, 2009; Fajardo and McIntire, 2010). Moreover, shifts from competition to facilitation at increasing stress have been demonstrated (e.g., Gross et al., 2013) despite exceptions and controversy (Maestre et al., 2009). Evidence also exists regarding the role of facilitation in milder environments (Holmgren and Scheffer, 2010; Granda et al., 2012), which brings about the broad prevalence of positive interactions and makes it clear that their effects on species coexistence, and thus on community diversity are likely wider than initially expected (Holmgren and Scheffer, 2010; McIntire and Fajardo, 2014).

The benefits of positive interactions on species composition have been addressed in a number of studies at local (Choler et al., 2001; Maestre et al., 2003; Cavieres and Badano, 2009) and, more rarely, regional or global scales (Valiente-Banuet et al., 2006; Cavieres et al., 2014). Cavieres et al. (2014) demonstrated in an extensive study using data sets across five continents that facilitation on cushion-dominated communities does not only enhance local but also global diversity, being as important as climatic drivers for the diversity of alpine ecosystems. If facilitation can have positive effects on species diversity, the opposite has also been demonstrated for aquatic organisms in stream mesocosms, where changes in species diversity altered the probability of positive species interactions, resulting in disproportionately large changes in the functioning of the study ecosystem (Cardinale et al., 2002). In plant communities, to our knowledge, this influence of species diversity on facilitation has rarely been addressed, although facilitation has been found to increase the phylogenetic diversity of the community (Valiente-Banuet and Verdu, 2007). Moreover, studies showing facilitation when strong niche overlap is present (Fajardo and McIntire, 2011), should shift our way to understand the species interactions. All these evidences demonstrate that facilitation is a ubiquitous driver of species diversity.

Global change impacts have been shown to be mitigated by facilitative interactions, including amelioration of climatic stress (Soliveres et al., 2011), reduced invasibility of communities by alien species (Bulleri et al., 2008) and increased survival, colonization or growth in habitats subjected to changes in land use (Gimeno et al., 2012). These processes allow for subordinate species, rare species or species with a low capacity to tolerate stress to survive thanks to the reduction of the environmental disturbances or intensity of the abiotic stress or predation (Hacker and Gaines, 1997). As a result, the biotic effects of nurse species should be combined with the nature and extent of environmental change to explain global patterns of species coexistence and to predict the effects of global change.

## Functional Traits, Phylogenetic Relatedness, and Community Assembly

Ecological differences among species are based upon their functional traits, which are expected to provide niche and fitness differences (HilleRisLambers et al., 2012; Kraft et al., 2015). Some key functional trait differences between plant species that stabilize coexistence by niche partitioning include differences in rooting depth, phenology, responses to environmental gradients such as light or pH and the specificity of the interactions with host-specific pathogens (Grubb, 1977; Rathcke and Lacey, 1985; Liu et al., 2012). Traits related to fitness differences are often associated with the ability to deplete a shared limiting resource (Harper, 1977; Tilman, 1987), which can be for instance height and size in light-limited environments, or root density and the efficiency to acquire nitrogen and phosphorous in poor soils (Ojeda et al., 2010; Hill et al., 2011).

Trait-based predictions of future changes in biodiversity can be carried out by identifying the functional mechanisms that generate niche partitioning and fitness differences (Adler et al., 2013). Many environmental changes involve altered supply of limiting resources. In the case of nitrogen availability, for example, direct supply coming from N deposition is favoring non-N fixers over N-fixers, grasses over legumes, and C3 grasses over C4 grasses (Reich et al., 2001; Stevens et al., 2004; Wookey et al., 2009). Some responses to global change may be more difficult to predict because they involve change in both niche and fitness differences between species. A clear example is the change on plant phenology due to increasing temperatures. Hotter days during spring are advancing the timing of flowering and leafing (Peñuelas and Filella, 2001; Wolkovich et al., 2012), but at the same time, hotter days during summer for some ecosystems such as temperate prairies are splitting species toward an earlier and a later phenology community (Sherry et al., 2007). Because earlier activity is associated with a fitness increase (Verdú and Traveset, 2005), we can expect that species advancing their phenology faster will exhibit a fitness advantage, which could destabilize coexistence. However, separating the temporal niche into two contrasted phenologies will act as a stabilizing mechanism. Whether species coexistence is maximized or species with earlier phenologies are favored will depend on (i) which phenological change is dominant and (ii) how strong these phenological changes link to niche and fitness differences. Interestingly, climate change has modified the phylogenetic pattern of temperate fields, wetlands, and deciduous forests in the last 150 years (Willis et al., 2008) reducing the abundance and presence of those clades that could not adjust flowering phenology in response to temperature changes. Because flowering time correlates with species fitness (Godoy and Levine, 2014), it is likely that the patterns of exclusion are due to changes in fitness differences between clades.

The niche occupied by a species is defined by several functional traits in response to simultaneous stressors operating at different temporal and spatial scales, referred to as the multidimensional niche (Hutchinson, 1957). Despite its intrinsic complexity, this multifunctional information should be incorporated into models that forecast future species distribution in response to climate change (e.g., Kearney and Porter, 2009). The difficulties associated to the notion that several traits are involved in species coexistence have moved researchers to look for other approximations that can simplify this complexity. Because phylogenies reflect the evolutionary history of competing species and at least in part their ecological capabilities, it is expected that species phylogenetic relatedness informs on the main ecological process involved in the assembly of the community (Ackerly, 2003). The use of coexistence theory is refining the common expectation from classic niche theory that competitive exclusion leaves coexisting species more evenly spaced across the phylogeny than expected by chance from the regional species pool because closely related species tend to share a similar niche (Webb et al., 2002). Mayfield and Levine (2010) suggested that phylogenetic relatedness may also reflect differences in competition among species, with competitive exclusion leaving coexisting species more phylogenetically clustered than expected by chance. Mayfield and Levine (2010) concluded that competition could have a contrasting role for the phylogenetic structure of communities and that the outcome can be predictable with a mechanistic understanding of how phylogeny determines the niche and fitness differences between competitors. This theoretical explanation, albeit simplistic, can contribute to detangle mixed results (clustering and overdispersion) from previous work on phylogenetic competition experiments (Duncan and Williams, 2002; Maherali and Klironomos, 2007; Violle et al., 2011; Allan et al., 2013; Bennett et al., 2013; Narwani et al., 2013), and it can serve also to understand why random phylogenetic patterns as well as closely related species coexist together in many natural communities (Godoy et al., 2014). For instance, a puzzling finding in many tropical forests is the substantial contribution of a small number of species-rich plant genera to the total pool of species (the so called species swarms). In the case of the understory shrubs of the genus *Psychotria* in Panama, one of the scant ecological studies of these species swarms, congeners were found unlikely to exclude one another because resource availability was determined largely by asymmetric competition with the overstorey since within the understory *Psychotria* shrubs had similar competitive abilities (Sedio et al., 2012).

Functional traits are being increasingly considered for understanding climate change impacts by their inclusion in dynamic global vegetation models (DGVMs). DGVMs are powerful tools to test ecological theories and they are actually incorporating new concepts arsing from community ecology and coexistence theory (Scheiter et al., 2013). Despite increasing refinements there is a lack of a comprehensive analysis of the direct impacts of trait variation on global vegetation distribution and dynamics. Results by van Bodegom et al. (2013) have shown a great predictive ability of these models when they account for just a few relevant traits. We argue that even higher predictive ability could be achieved if intraspecific trait variability is included, as discussed in Valladares et al. (2014b).

## Intraspecific Trait Variability

Species functions have been primarily defined on the basis of the mean values of their functional traits (Figure 1A), ignoring the extensive intraspecific variation typically found for most traits (Figure 1B). In fact, the contribution of intraspecific trait variability to trait-based coexistence theory has been underestimated over decades (Albert et al., 2010; Mitchell and Bakker, 2014). As a result of this research gap, an increasing number of studies have underlined the importance of incorporating information of intraspecific trait variation as a driver of species coexistence and community dynamics (Bolnick et al., 2011; Courbaud et al., 2012; Figure 1B). A study from forests in the southeastern of the United States revealed that the variation among the individuals within the study populations generated different distributions and responses to the environment among species, while the mean values for the corresponding populations did not differ (Clark, 2010). Lichstein et al. (2007) investigated the potential for intraspecific individual variation to maintain species coexistence through the use of a two species model assigning to each species a random independent competitive ability. These simulations showed that if the density of individuals competing for an open area is high, species with a large variance in competitive ability are favored, whereas the reverse is true if density is low. If there is an interspecific mean-variance competitive ability trade-off (e.g., one species competes against a second species that has a lower mean but a higher variance in individual competitive ability), stable coexistence can be expected over a range of intermediate densities. A superior vs. an inferior species (e.g., different means but the same variance in individual competitive ability) are expected in the absence of such a trade-off, and intraspecific variation would blur differences among species and the dynamics would follow the neutral case expectations. Even though Lichstein et al. (2007) showed that intraspecific variation can facilitate coexistence, they consider that it could play only a minor role for maintaining diversity in many real communities, which needs to be further explored.

Several studies have shown changes in the intraspecific variation of plant functional traits in response to new environmental conditions and new selection pressures resulting from global change drivers. For example, not only species turnover but also, and highly significant, intraspecific trait variability was found to be key in the functional response of alpine plant communities to drought (Jung et al., 2014).

An important source of intraspecific trait variability with key implications for population differentiation and local adaptation is phenotypic plasticity (Valladares et al., 2014b). Phenotypic plasticity can be defined as the ability of a genotype to show variable phenotypes in response to different environments (Garland and Kelly, 2006; Valladares et al., 2007). It has been widely recognized as a mechanism to cope with spatial and temporal heterogeneity, thereby avoiding migration or extinction of organisms under highly variable or increasingly distressed conditions (Matesanz et al., 2010; Nicotra et al., 2010). Jung et al. (2010) studying the role of the intraspecific trait variation on species assembly in grassland communities distributed along a flooding gradient found evidence that plasticity in resource use at the population level was an important mechanism of niche differentiation among plants. The promotion of species coexistence through resources partitioning have also been supported by Callaway et al. (2003), Miner et al. (2005), and Ashton et al. (2010). The lack of consistent patterns across lineages and geographical ranges together with the scarcity of sound empirical studies is challenging the inclusion of phenotypic plasticity in species distribution models used to forecast biodiversity under global change scenarios (Valladares et al., 2014b). As already noted by Pearman et al. (2010), species distribution models improve their results when incorporating within-species variation.

However, and despite the importance of intraspecific trait variability, its inclusion in trait-based coexistence theory remains a topic open to discussion (Kraft and Ackerly, 2009; Lake and Ostling, 2009; Bolnick et al., 2011). Albert et al. (2011) proposed a guideline on when intraspecific trait variability should be taken into account in ecological studies. The sequential steps of this guideline were: (1) whether the study explicitly encompasses intraspecific trait variability, such as evolutionary studies interested in trait or niche evolution; (2) the spatial scale of the study, with local studies typically more concerned with intraspecific trait variability; (3) the way the study species were chosen, i.e., species (e.g., few focal species) vs. site (e.g., all species within community) centered studies; in the former intraspecific trait variability is central, while in the latter the species turnover effect could be higher than the intraspecific trait variability effect. To decide upon the importance of including intraspecific trait variability in the case of site-centered studies, one more question still needs to be answered, (4) whether the study is interested in effect or response traits; in the latter case intraspecific trait variability is clearly central, while in the former case it might be omitted. Intraspecific trait variation seems appropriate to unify classic coexistence theory and evolutionary biology with recent trait-based approaches. For example, including this variation source in a hierarchical Bayesian model rendered accurate and realistic predictions and avoided some of the criticisms associated with some trait-based community assembly models (Laughlin et al., 2012).

## Environmental Heterogeneity and Dynamic Mosaics

Spatial heterogeneity can have a strong impact on species coexistence (Figure 3). In heterogeneous environments, species can be segregated in space according to their niche preferences (e.g., resource requirements). Classical examples include for instance differences in which chemical forms of nitrogen compounds are uptaken by tundra species (McKane et al., 2002). This prediction, inspired by the classical niche theory and contrary to the neutral theory, has been proven to promote coexistence in tropical forests over a wide sample of biogeographic conditions (Brown et al., 2013). Model simulations reveal the potentially important role of heterogeneity and its complex and delicate interplay with dispersal in mediating long-term outcomes of species coexistence (Schreiber and Killingback, 2013). For instance, when resource-rich patches are formed by an engineering species, habitats for species with high dispersal capacities are provided, allowing a successful colonization by these other species and their eventual coexistence with the engineering species. This spatial self-organization phenomenon has been reported by Nathan et al. (2013) using a mathematical formulation. While many studies recognize that spatial heterogeneity promotes species diversity, high species diversity itself can also increase spatial heterogeneity for factors like light (each species canopy intercept light differently) or soil water and nutrients (each species explores below ground resources differently), which in turn could allow for more species to coexist by attenuating competition. There is thus a potential positive feedback loop between local and regional heterogeneity and species diversity (Nathan et al., 2013).

Spatial heterogeneity is particularly relevant for coexistence of sessile organisms like plants (Bolker et al., 2003), and its effect on plant performance can vary according to the life history of the individuals as well as to the particular spatial scale considered. For instance, species colonization in Mediterranean forests has been found to depend on the identity of the dominant species at regional scales during the seed-seedling transition, while it was found to depend on local heterogeneity once seedlings had emerged (Granda et al., 2014). Further, the role of spatial heterogeneity can be strong when coexistence is quantified at scales larger than those perceived by the organisms, e.g., when coexistence of species locally segregated by fine-grained heterogeneity is determined at regional scales. This role of spatial heterogeneity when coexistence is assessed at a coarser grain than that perceived by the organisms has been shown to explain coexistence in the case of microorganisms dwelling in patchy soils (Porter and Rice, 2013). Different scales of heterogeneity can also explain vegetation patterns in Mediterranean ecosystems where dominance of one species at local, patch level is compensated by the co-occurrence of close-by patches dominated by different species. Moreover, in these ecosystems dominated by a few tree species the juveniles have been shown to recruit preferentially in non-conspecific stands, generating dynamic mosaics within a landscape where patches dominated by each species promote species turnover over time (Granda et al., 2012, 2014; Galiano et al., 2013). In addition, metacommunity approaches explicitly link local and regional community dynamics. Gilbert and O’Connor (2013) also highlighted that the metacommunity theory allows scaling up from community-level processes to regional patterns of species distribution and dynamics. Despite their potential for exploring the influence of regional processes, such as dispersal and habitat configuration, on local abundances and occurrences few studies incorporate metacommunity dynamics into a global change framework (Gilbert and O’Connor, 2013) due to the challenge of determining the effects of global change on processes at different scales and to account for their synergy (O’Connor et al., 2012). However, metacommunity models can appropriately guide research on how climate change alters specific local and regional processes and the feedbacks between them determining coexistence (Anderson et al., 2015). In turn, empirical research can identify important gaps in metacommunity approaches (Gilbert and O’Connor, 2013).

Equally important for the maintenance of coexistence is the heterogeneity in time, with an influence on natural communities also variable depending upon the temporal scales. Temporal fluctuations can stabilize coexistence via storage effect (Chesson, 2000), when inter-annual variation in climate or resource availability favors alternatively one group of species over the others (e.g., Zavaleta et al., 2003). Not only inter-annual but also seasonal variability contributes to fluctuating resources that increase the number of coexisting species in different systems (Angert et al., 2009; Shimadzu et al., 2013). Oscillations at the population level can further be a consequence of species interactions with shared resources (i.e., endogenous compensatory dynamics, González and Loreau, 2009), when the species with a saturating growth response generates cycles of the resource. As a result, community dynamics are ensured by both species interactions and different responses to the fluctuating environment.

Disturbance in space and time is important for species coexistence in environments that are relatively homogeneous so it breaks at least temporarily this homogeneity. Such a disturbance regime becomes key for competition-colonization trade-offs (Cadotte, 2007). These trade-offs are the basis for the intermediate disturbance hypothesis, which states that diversity of competing species is maximized at intermediate frequencies or intensities of disturbance or environmental change (Bongers et al., 2009). However, and despite the abundant and interesting research yielded with this hypothesis, a revision of its current theoretical and empirical foundations suggests that it should be abandoned (Fox, 2013). Empirical studies only rarely find the predicted humped diversity-disturbance relationship and the three theoretical mechanisms claimed to produce this relationship are logically invalid (Fox, 2013). Originally created to explain patterns of diversity in tropical forests, its explanatory value is poor even in this ecosystem as shown in an extensive review (Bongers et al., 2009). While diversity did peak at intermediate disturbance levels little diversity variation could be explained outside dry forests since disturbance had less influence on species richness patterns in wet tropical rain forests than typically assumed (Bongers et al., 2009).

Two fundamental drivers of environmental change for plant communities are long-term increases in soil resource availability and grazing pressure (Adler et al., 2001; Laliberte et al., 2013). These changes are expected to produce profound changes in diversity and species composition, and one expects that in general they reduce diversity by exclusion. For those species that coexist thanks to heterogeneous environments, an increase in resource supply can homogenize differences between patches. With a more homogenized environment the likelihood of coexistence is smaller because this tends to favor the species that can better exploit a single environment. For instance, Southon et al. (2013) showed that across the UK, nitrogen deposition is reducing diversity in the heathlands with a few species dominating across regions. Similar results were obtained in a manipulative experiment of a Californian grassland by Zavaleta et al. (2003), where a homogenization of the environment caused by increases in nitrogen deposition decreased the number of coexisting species at patches that were not subjected to any degree of disturbance. Similar losses of diversity can occur when the degree of disturbance is too high, because only a few species will be able to survive in such stressful environments, as it is occurring with the loss of plant, bird, and mammal diversity in intensified rural landscapes (Flynn et al., 2009).

Another functionally important aspect of heterogeneity is the increased frequency and intensity of extreme climatic events caused by climate change. These perturbations are leading to species-specific mortality, changing competitive ability differences among species, reducing the abundance of the dominant species, and, therefore, changing the long-term population and community trends (Thibault and Brown, 2008). Holmgren et al. (2006) showed strong cascade effects from species responses to community-level changes in arid and semi-arid ecosystems worldwide after changes associated to El Niño Southern Oscillation (ENSO). Limited capacity of native communities to maintain their structure and function after extreme climatic events has been shown to favor the invasion process (Diez et al., 2012), and changes in the dominance of native species within communities due to different growth responses and recovery patterns during and after extreme droughts have also been suggested (Cavin et al., 2013; Granda et al., 2013).

**FIGURE 4:**
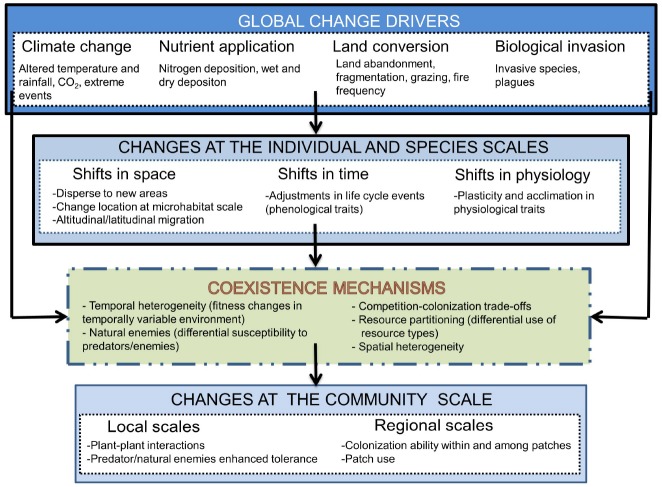
**Global change drivers affect coexistence mechanisms in a number of ways, at various levels of biological organization (from individuals to species) and at various spatial and temporal scales.** Individual fast responses to environmental change co-occur with alterations in species interactions, resource use and many other changes that interactively affect species coexistence. Changes observed at the community level are thus resulting from the direct effect of global change drivers on both coexistence mechanisms and individual species responses to changes in these drivers.

All drivers of global change are expected to exert an important effect on coexistence mechanisms and, therefore, to change the outcome of species interactions (Figure 4). Anthropogenic environmental and climatic changes for example are dramatically varying the resource supply at multiple spatial and temporal scales (Matesanz and Valladares, 2014; Valladares et al., 2014a). This variation is important because the stability of the resources affects species’ abilities to capture them (Nathan et al., 2013; Parepa et al., 2013). Overall, this variation is leading to extinction rates that are significantly higher than what would be expected from the fossil record (Bálint et al., 2011; Barnosky et al., 2011). However, increased diversity is also being observed at global scales in certain ecosystems such as alpine grasslands (Cavieres et al., 2014).

## Biological Invasions, a Coexistence Lesson in a Changing World

Human activity is transporting, either voluntarily or involuntarily, thousands of species through long distance and although many species fail to establish, some species become new elements of the local biota (Hulme, 2009). Coupled to the global phenomena of biological invasions, a whole body of literature has emerged in ecology to understand the mechanisms by which invasions occur (reviewed in Catford et al., 2009). Biological invasions therefore represent a good example to relate recent advances in coexistence theory to the effect of global change on natural ecosystems, as most of the factors driving invasion can be better understood within a framework of niche and fitness differences. MacDougall et al. (2009) have shown that the mechanisms driving invasion and the maintenance of species diversity are two sides of the same coin. Invasion and exclusion of the native community occur when the fitness advantage of the exotic species overwhelms the stabilizing niche differences with the resident community. Invasion and coexistence with the native residents occur when exotic species are able to enter into the system at low relative abundance because of their niche differences with respect to the native residents but these niche differences prevent them from excluding native species while becoming abundant (MacDougall et al., 2009; Figure 3).

Understanding whether invaders benefit from either fitness differences or niche differences, or both is crucial if we aim to control and eliminate invasive species, a common target of restoration and conservation programs. Prior conceptual and experimental work using trait-based approaches has argued for the concept of limiting similarity to accomplish successful restoration actions (Emery, 2007; Funk et al., 2008). The underlying idea is that functional traits reflect species’ niche. Nevertheless, this approach can be limited for the two following reasons. First, native species may not possess the functional characteristics needed to fill the same niche as the invaders. Second, functional traits can reflect both species niche and fitness. In this latter case, using native species with similar characteristics to those of the invader may not turn into the result desired. A clear example focusing again on phenology illustrates this problematic issue. In a California grassland, Godoy and Levine (2014) have found that differences in phenology promote both stabilizing niche differences between annual plant competitors and fitness differences between them. Fitness advantages were greater on later phenology species. Importantly, the fitness advantage of later phenology species overwhelmed the stabilizing effect of phenological offset competitors allowing later invaders to outcompete earlier native communities and native residents to outcompete earlier-phenology invaders (Fridley, 2012). Overall, these results highlight two important messages. First, by focusing only on functionally dissimilar native communities, invasive species with early phenology can be eliminated from the system. Second, some aims of restoration cannot be accomplished. In this example, later invasive annual species cannot be repelled with native annual communities. Perhaps, shrub and tree encroachment could eliminate these later invasive species by shading, which will probably reduce their fitness, but this action is in conflict with the maintenance of a grassland system.

Different drivers of global change can modify niche and fitness differences between invaders and resident communities, and hence modulate the impact of biological invasions (Table 1). For instance, climate change can increase invader’s population size presumably due to a relative increase in fitness with respect to the native community during periods of climatic amelioration such as increasing temperatures for thermophilous plants (Walther et al., 2002). Climate change, through extreme events such as heat waves, hurricanes, flood, and drought is also expected to promote invasion success (Diez et al., 2012). In general, extreme climatic events produce simultaneously a reduction of the fitness of the native residents and an increase of the fitness of the invaders thanks to a resource pulse. This combination occurs when the stress tolerance of invaders to abiotic factors is higher (Diez et al., 2012; Gioria and Osborne, 2014). For example, non-native vines benefited more than native vines from the full-exposed sun conditions derived from wind-driven tree canopy loss after Hurricane Andrew in Florida in 1992 (Horvitz et al., 1998; see Diez et al., 2012, for other examples).

New niche opportunities favoring invasion come often from anthropogenic changes (Shea and Chesson, 2002), which are ubiquitous components of global change. In general, invasive species maintain self-sustaining populations and disperse through disturbed habitats such as roadsides, railways, human-modified rivers and abandoned cultures that become semi-natural grasslands. The idea that the invader’s niche is linked to perturbation, is common (Lake and Leishman, 2004; Pauchard and Alaback, 2004) to the extent that invaders are seen as passengers more than drivers of the habitat changes (MacDougall and Turkington, 2005). The limitation to native species for exploiting these new niches created by anthropogenic changes can come from different functional, ecological and evolutionary sources (Matesanz and Valladares, 2014). For example, water irrigation is creating a new niche in Spanish Mediterranean ecosystems with minimized drought during summer (Godoy et al., 2009). Native species are not able to exploit this niche because of their evolutionary constraints to display mostly a spring phenology. However, invasive species that evolved in tropical environments display summer phenology matching the time frame of resource availability (Godoy et al., 2009). This fact can increase the overall number of species that can be found in a particular ecosystem (Knops et al., 1999), because exotic species do not produce any harm to the native community, but also increases the risk of invasion since rapid evolution to more drought adapted phenotypes can occur easily.

## Impacts of Global Change on Species Coexistence in Pauci-Specific Systems: The Case of Mediterranean Forests

Despite being within a biodiversity hotspot, Mediterranean forests are typically dominated by only two-three tree species, particularly in dry, continental areas (di Pasquale et al., 2004). Empirical studies aimed at characterizing mechanisms of species coexistence in Mediterranean forests are scarce. These generally include: (i) differential species responses to environmental stress, (ii) dispersal patterns and, (iii) spatial heterogeneity, which, coupled with facilitation, are recognized as the main mechanisms promoting coexistence (Gómez-Aparicio, 2008; Granda et al., 2012; Pérez-Ramos et al., 2012a, Galiano et al., 2013). Pérez-Ramos et al. (2012b) found within and among species differences through plant ontogeny arising from species differential responses to microhabitat heterogeneity and seed size variation in a mixed-oak forest of southern Spain, further confirmed by Granda et al. (2014) in continental Mediterranean forests. Galiano et al. (2013) also focused on regeneration patterns of oak species in a pine-dominated forest of north-east Spain, where pine mortality was not compensated by its regeneration, suggesting vegetation shifts to oak-dominated forests if the intensity and frequency of extreme droughts keep increasing. In addition to these and other studies addressing coexistence, we suggest that more research is needed to improve our understanding of the specific mechanisms involved, such as those common in species rich ecosystems (i.e., tropical forests) that have been rarely identified in pauci-specific ones (i.e., Mediterranean forests where a few engineering species dominate the canopy). We suggest that negative density dependent processes, including predation, herbivory or pathogen infection could also modulate coexistence in Mediterranean ecosystems by promoting the recruitment away from parent trees and freeing potential colonization areas for other species (Granda et al., 2014). So far, non-random patterns of pathogen infection (predictable by both abiotic and, particularly, biotic factors as tree and shrub species presence) and their role in plant communities have been described in southern Spain (Gómez-Aparicio et al., 2012). However, further research is needed to test whether negative density dependence could promote species coexistence in the Mediterranean region if, for example, infection of the most common species favors the establishment of other species in accordance with the Janzen–Connell hypothesis (Janzen, 1970; Connell, 1971). Moreover, the alteration of these coexistence mechanisms under ongoing global change should be better described to be able to predict future directions in forest dynamics.

Despite the loss of diversity in rich ecosystems being a crucial concern for ecologists and conservationists, the ecosystems that are perhaps more endangered by global change drivers are those containing a low number of species that contribute significantly to its functioning and productivity.

Recent studies have highlighted that the resilience of a system, (i.e., the ability of a community to respond to global changes) depends on the functional diversity of a community rather than its species richness (Diaz et al., 2007). In pauci-specific ecosystems, species loss may have serious consequences for the functional diversity, collapsing the system when the species lost cannot be replaced by another species with similar function. In other words, the limited functional redundancy that is mathematically possible in a pauci-specific ecosystem makes them more vulnerable to species loss at least from a probabilistic point of view. This is the case of many Mediterranean forests, where coexisting species tend to present strong dissimilarities in their traits. Instead, other ecosystems with a higher number of species may show similar functional diversity, indicating that trait values among species are also similar. In these sites, functional redundancy may buffer against the impact of climate change on the local species pool as shown by Gallagher et al. (2012).

Most drivers of global change such as increased aridity, pollution, land use change and increased fire risk, all of them already exerting great pressures on Mediterranean ecosystems (Doblas-Miranda et al., 2014; Valladares et al., 2014a), are presumably going to reduce species fitness up to the point to limit their survival under these new conditions (Matesanz and Valladares, 2014). At least two scenarios emerge as alternatives to the simplistic expectation of species gradual extinction under such increase of environmental pressure: (i) coexistence is maintained by changes in species interactions (increased role of facilitation, complex multi-species interactions reinforced), which can buffer the pressure, and (ii) within species functional variability could compensate for the limited number of species making up the community. Knowledge on factors influencing the occurrence of these two alternatives and on their implications is still very limited to assess their potential for counteracting the negative impacts expected from the increased environmental pressure.

## Conclusion

There is an urgent need to understand how different drivers of global change differentially but simultaneously impact ecosystems and which are the corresponding magnitude and direction of the changes in species interactions and coexistence. Recent developments of ecological theories are improving the forecast of these changes but more empirical data are needed for a solid theory of the mechanisms driving species coexistence.

There are three main empirical approaches to the study of community assembly: experimental manipulations of the abiotic or biotic environment, assessments of trait-phylogeny-environment relationships, and quantification of frequency-dependent selection and population growth. Each approach alone is not strong enough to reveal which niche axes and which traits determine the outcome of competition, the extent of facilitation and the eventual structure and dynamics of the community. Thus, only the combination of these three approaches can significantly contribute both to conceptual ecology and to guidelines for ecosystem management under global change (HilleRisLambers et al., 2012). Nonetheless, the combination of the three in a single research project requires an enormous effort that sometimes is unjustified. The degree of resolution would depend on the research aim. For instance, if the question is related to how species are precisely responding to a combination of different global change drivers (e.g., an increase in precipitation or aridity, an increase in nitrogen deposition, or an increase in grazing) then to study how these drivers affect species fitness could be enough. However, if the question relates to how specific species responses translate to community dynamics, then it is also necessary to study niche differences among species to know the outcome of species interactions. While the amazing richness of ecosystems like tropical forests have attracted fruitful research and theories on species coexistence, there is much that can be learnt from pauci-specific communities where the value of each single species is large and where the functional variability within each species becomes key to understand species interactions and eventual community responses to global change. In both research and conservation activities, we have to move from species coexistence to the coexistence of genotypes, paying more attention to the functional variability existing within each species.

## Author Contributions

All authors jointly developed the concept of this paper, contributed with ideas and information and wrote the manuscript.

### Conflict of Interest Statement

The authors declare that the research was conducted in the absence of any commercial or financial relationships that could be construed as a potential conflict of interest.
